# Starch and starch hydrolysates are favorable carbon sources for Bifidobacteria in the human gut

**DOI:** 10.1186/s12866-015-0362-3

**Published:** 2015-03-01

**Authors:** Songling Liu, Fazheng Ren, Liang Zhao, Lu Jiang, Yanling Hao, Junhua Jin, Ming Zhang, Huiyuan Guo, Xingen Lei, Erna Sun, Hongna Liu

**Affiliations:** Key Laboratory of Functional Dairy, College of Food Science & Nutritional Engineering, China Agricultural University, Beijing, 100083 China; Higher Institution Engineering Research Center of Animal Product, Beijing, 10083 China; Beijing Laboratory for Food Quality and Safety, Beijing, 10083 China; Department of Animal Science, Cornell University, Ithaca, NY 14853 USA

**Keywords:** Bifidobacteria, Starch, Nutrient-niche, Adaptation, Gemomics

## Abstract

**Background:**

Bifidobacteria are key commensals in human gut, and their abundance is associated with the health of their hosts. Although they are dominant in infant gut, their number becomes lower in adult gut. The changes of the diet are considered to be main reason for this difference. Large amounts of whole-genomic sequence data of bifidobacteria make it possible to elucidate the genetic interpretation of their adaptation to the nutrient environment. Among the nutrients in human gut, starch is a highly fermentable substrate and can exert beneficial effects by increasing bifidobacteria and/or being fermented to short chain fatty acids.

**Results:**

In order to determine the potential substrate preference of bifidobacteria, we compared the glycoside hydrolase (GH) profiles of a pooled-bifidobacterial genome (PBG) with a representative microbiome (RM) of the human gut. In bifidobacterial genomes, only 15% of GHs contained signal peptides, suggesting their weakness in utilization of complex carbohydrate, such as plant cell wall polysaccharides. However, compared with other intestinal bacteria, bifidobacteiral genomes encoded more GH genes for degrading starch and starch hydrolysates, indicating that they have genetic advantages in utilizing these substrates. *Bifidobacterium longum* subsp. *longum* BBMN68 isolated from centenarian’s faeces was used as a model strain to further investigate the carbohydrate utilization. The pathway for degrading starch and starch hydrolysates was the only complete pathway for complex carbohydrates in human gut. It is noteworthy that all of the GH genes for degrading starch and starch hydrolysates in the BBMN68 genome were conserved in all studied bifidobacterial strains. The *in silico* analyses of BBMN68 were further confirmed by growth experiments, proteomic and real-time quantitative PCR (RT-PCR) analyses.

**Conclusions:**

Our results demonstrated that starch and starch hydrolysates were the most universal and favorable carbon sources for bifidobacteria. The low amount of these carbon sources in adult intestine was speculated to contribute to the low relative abundance of bifidobacteria.

**Electronic supplementary material:**

The online version of this article (doi:10.1186/s12866-015-0362-3) contains supplementary material, which is available to authorized users.

## Background

Bifidobacteria are autochthonous inhabitants of human gut and their presence is considered as an important indicator of healthy microbiota. Bifidobacteria are dominant in the infant gut, but they only account for 3-6% of the adult fecal flora [1]. Their abundance was influenced by the ability to adapt to the human intestinal environments. According to Freter’s nutrient-niche theory, individual species in human microbiota have preference to one or a few of the nutrients in their niche and individual population sizes are determined by the available concentration of their preferred nutrients [2-4]. In the colon, only host glycans and diet-derived polysaccharides are available to bifidobacteria [5,6]. Therefore, the ability of bifidobacteria to use these carbohydrates is an important factor determining their abundance within the intestinal microbiome [3].

Genomics have proven to be a very powerful tool to predict carbohydrate-acquisition strategies of bifidobacteria. Genomic analysis showed that *Bifidobactereiu bifidum* PRL2010 and *Bifidobacterium longum* subsp. *infantis* respectively targeted host-derived glycans and human milk oligosaccharides (HMO) [7,8]. Kaoutari et al. analyzed the glycoside hydrolase and polysaccharide lyase profiles of a representative microbiome of human gut, providing an integral prediction on their carbohydrate substrates [9]. *Bifidobacterium longum* subsp. *longum* BBMN68 was isolated from the faeces of a centenarian [10]. The sequence analyses of the BBMN68 genome revealed a high proportion of genes for carbohydrate transport and metabolism [11]. Meanwhile, a survey showed that the crude corn and other foods was the main staple food for the centenirian [12]. Therefore, it was speculated that the high intake of starch and dietary fibre will endow BBMN68 strong ability to utilize these carbohydrate.

In the present study, the carbohydrate utilization system of bifidobacteria was explored by comparing their GH profiles with that of the representative microbiome. Predicted metabolic pathways of BBMN68 were constructed and were further confirmed *in vivo* by proteomic and RT-PCR analysis. Our results showed that starch and starch hydrolates were the favorable carbon sources for bifidobacteria. Starch is a highly fermentable substrate and has been shown to exert beneficial effects by increasing bifidobacteria and/or being fermented to short chain fatty acids [13]. However, the poor availability of these carbohydrates [14,15] in the human gut is speculated to be an important reason for their relatively low abundance of bifidobacteria. To our knowledge, this was the first report about the genetic interpretation for the low relative abundance of bifidobacteria in adult gut.

## Results

### The distribution of genes encoding glycoside hydrolases (GHs)

**Figure 1:**
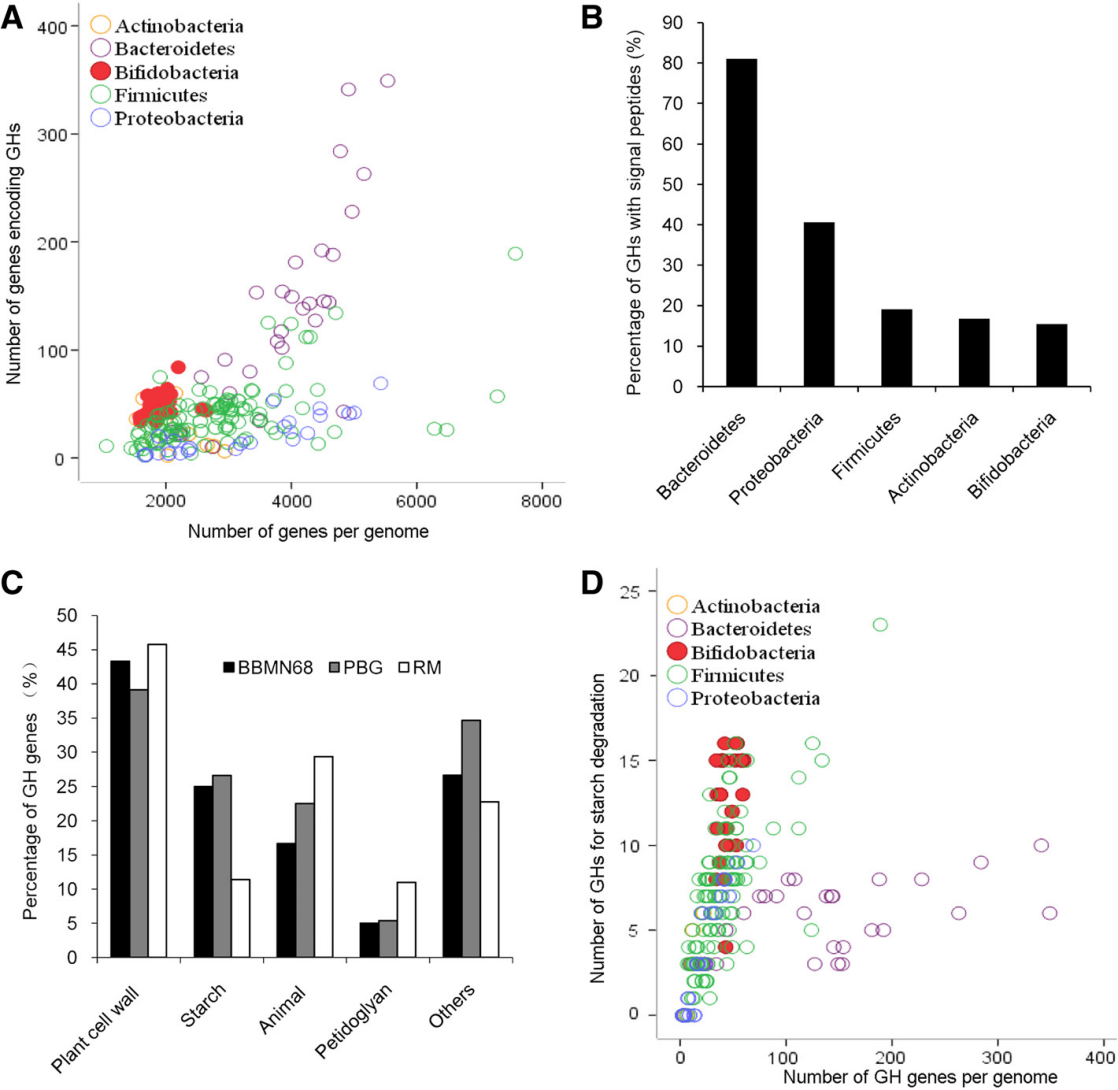
**Glycoside hydrolases in bacterial strains. (A)** GH genes in bacterial genomes. **(B)** Percentages of GHs with signal peptides. **(C)** Predicted substrate categories of GHs. **(D)** GHs for degrading starch and starch hydrolysates in bacterial genomes.

The GH genes in 25 bifidobacterial genomes (Additional file 1) were compared with those in intestinal bacterial strains from a recently reported representative microbiome (RM) of human gut [9]. The number of GH genes in bifidobacteria was lower than that in some *Bacteroidetes* and *Firmicutes* strains, but higher than the other strains studied (Figure 1A). Compared with the other strains, the proportion of GHs with signal peptides is the lowest in bifidobacterial genomes (Figure 1B). For further analysis, a pooled-bifidobacterial genome (PBG) that contained the 25 bifidobacteria was built, and the substrates of the GHs in PBG were compared with those in RM (Figure 1C). In the PBG and RM, the highest percentages of GHs were predicted to target plant cell wall polysaccharides, *i.e.* 43% and 46% of the total GHs, respectively. However, the percentage of GHs targeting starch and starch hydrolysates in PBG was markedly higher than that in RM (27% in PBG vs. 11% in RM). Furthermore, we found that the number of GHs for degrading starch and starch hydrolysates was higher in almost all bifidobacterial strains than in strains of other genus (Figure 1D).

**Figure 2:**
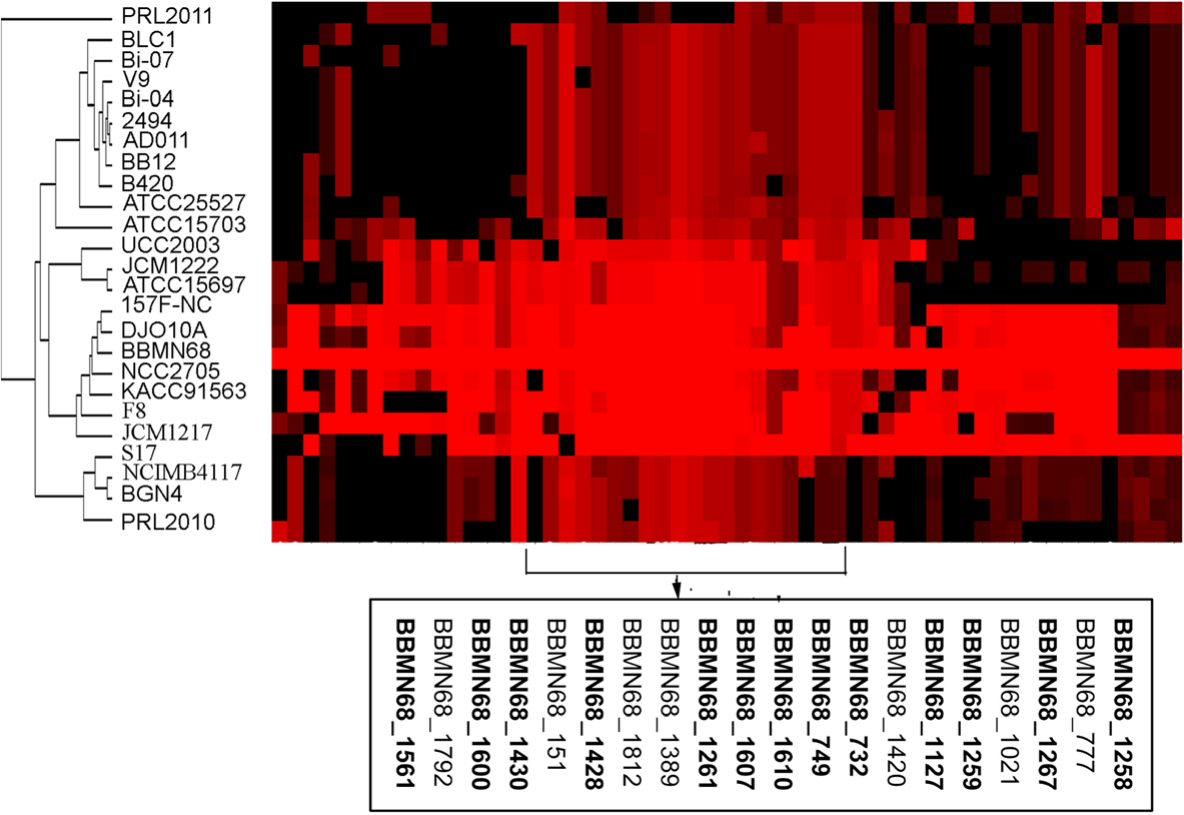
**Diversity of genes encoding GHs in bifidobacterial strains with reference to those in the BBMN68 genome.** Columns represent analyzed strains, which are identified by their code numbers. The color code varies from black to red and indicates absence, divergence or presence of a gene. The most conserved genes are shown in the bracket and the genes involved in starch degradation are in the black box.

We then choose BBMN68 as a model strain to further investigate the GHs of bifidobacteria. The BBMN68 genome contained 58 genes encoding glycoside GHs that were distributed in 25 families (Additional file 2). In all the proteins encoded by 58 genes, 13 are in GH13 family (targeting starch), and 9 are proteins in GH43 family (targeting plant cell wall polysaccharides). The substrates of the 58 GHs in BBMN68 shared the same distribution as PBG (Figure 1C). The GHs for plant cell wall polysaccharides and starch accounted for 43% and 25% of the total GHs, respectively. In order to compare the GH genes from BBMN68 with those from other bifidobacterial strains, a BLAST heatmap was constructed (Figure 2). Interestingly, all GH genes for degrading starch and starch hydrolysates were conserved in all bifidobacterial strains.

### Prediction of carbohydrate utilization in BBMN68

**Figure 3:**
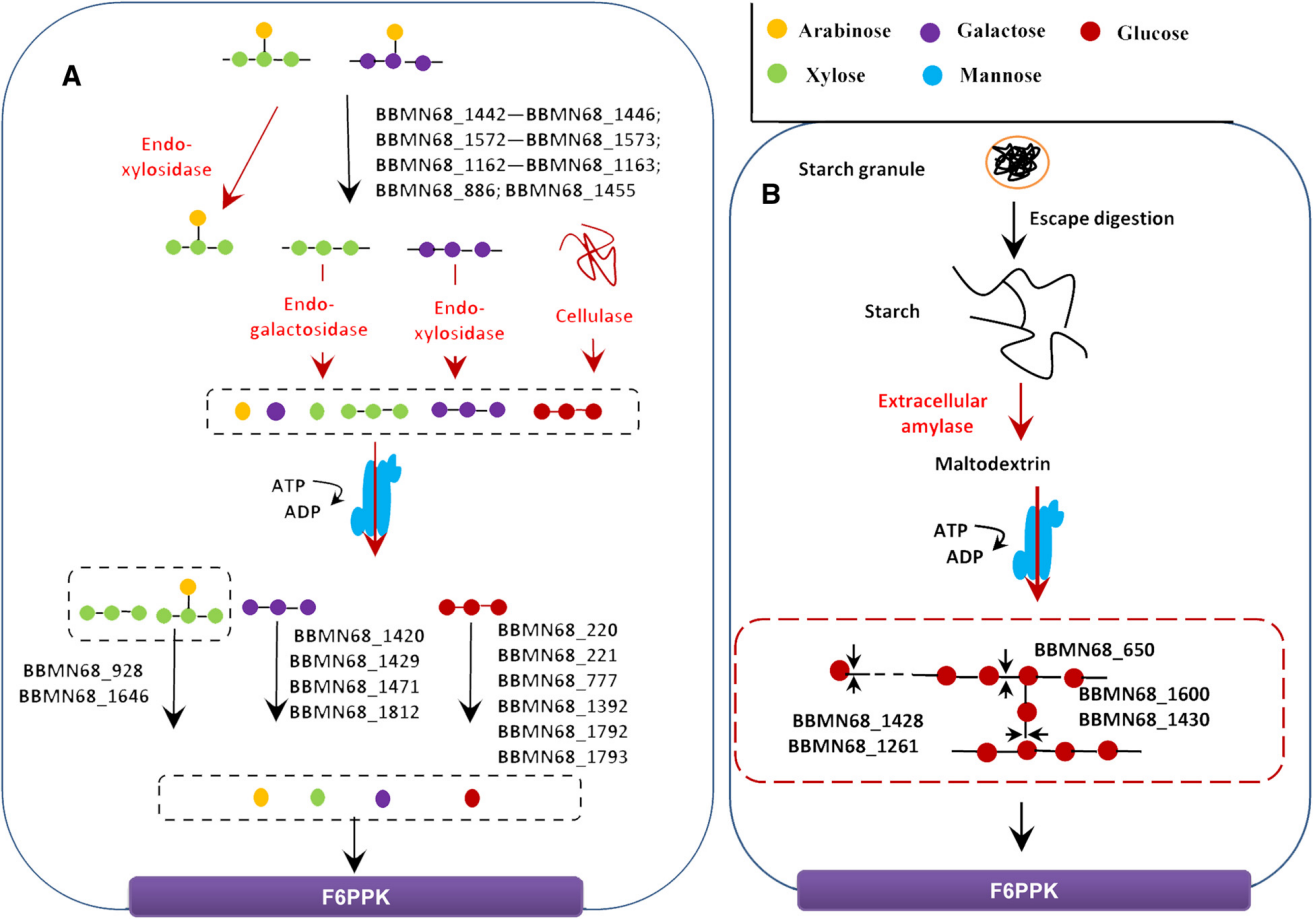
**Predicted pathways for carbohydrates metabolism of BBMN68. A**. Schematic representation of the metabolic pathway for plant cell wall polysaccharide. **B**. Schematic representation of the metabolic pathway for starch and starch hydrolysates. F6PPK: fructose-6-phosphate phosphoketolase pathway.

In order to acquire comprehensive information about the carbohydrates degradation, we constructed predicted pathways of carbohydrate metabolism inBBMN68. BBMN68 genome contained 6 GH genes, which are responsible for degrading starch and starch hydrolysates. These genes encoded alpha-amylase (BBMN68_650, BBMN68_1257), alpha-glucosidase *(BBMN68_1428, BBMN68_1261)* and alpha-1, 6-glucosidase (BBMN68_1600, BBMN68_1430). It is speculated that these enzymes could completely degrade starch to glucose (Figure 3). Furthermore, BBMN68 contained genes encoding GH13 family sucrose phosphorylase (*BBMN68_1267*), 4-alpha-glucanotransferase (*BBMN68_1259, BBMN68_1607*), which produce glucose 1-phosphate from maltodextrins in starch degradation V pathway [16,17]. Finally, pullulanase-like glycosidases involved in starch degrading [18], were also found in the BBMN68 genome (BBMN68_732, BBMN68_749, BBMN68_1610, BBMN68_1127). However, genes encoding extracellular amylase were not found in the genome of BBMN68. It was speculated that BBMN68 can’t directly utilize starch, since this strain does not produce an extracellular amylase and the chain of starch is too long to be transported into the cell. These results suggested that BBMN68 could only use starch hydrolysates as a substrate, such as maltodextrins (Figure 3) [15].

BBMN68 genome contained 9 GH43 genes and 3 GH51 genes, which encode enzymes for hydrolyzing the alpha-L-arabinosyl side chains of polysaccharides [19]. Interestingly, all of the 9 enzymes in GH43 and one in GH51 were located extracellularly or anchored to cellwall (Additional file 1: Table S1). As shown in Figure 3, the extracellular enzymes in GH43 and GH51 families can hydrolyze the alpha-L-arabinosyl side chains of polysaccharides from the plant cell wall, such asarabinoxylans. The arabinose derived from this process can then be used by BBMN68 or other members in the microbiota. However, further degradation of polysaccharides required enzymes from other gut microbiotal members, since there was no endo-enzyme for these substrates in the BBMN68 genome. Only after they were degraded by endo-enzymes, the products such as galactooligosaccharides, xylooligosaccharides or ara-xylooligosaccharides, would be transported into BBMN68 cells for further degradation by enzymes encoded by *BBMN68_1420*, *BBMN68_1471*, *BBMN68_1471*, *BBMN68_1812*, *BBMN68_928*, and *BBMN68_1646* (Figure 3). In addition, BBMN68 have potential to use incomplete hydrolysates of cellulose, since the genes for encoding beta-glucosidases (BBMN68_220, BBMN68_221, BBMN68_777, BBMN68_1392, BBMN68_1792, BBMN68_1793) were also found in the genome of BBMN68.

In addition to enzymes targeting starch or plant cell wall polysaccharides, there were also enzymes targeting animal glycans, peptidoglycans, fungal glycans, sucrose orfructans. BBMN68_930, BBMN68_1201 and BBMN68_1812 were annotated as beta-galactosidases, which are essential enzymes to degrade oligosaccharides in milk. The predicted substrates of BBMN68_151 were inulin or fructooligosaccharides, which are storage carbohydrates in some plants. BBMN68_215 and BBMN68_216 are enzymes classified into EC:3.2.1.24, which are predicted to hydrolyze the terminal, non-reducing alpha-D-mannose residues of alpha-D-mannosidic linkage in glycoproteins. BBMN68_99, BBMN68_222 and BBMN68_1202 are predicted to participate in the degradation of HMO or mucin-derived oligoshaccharides [8,20].

### Verification of starch and starch hydrolysates degrading pathway activity

**Figure 4:**
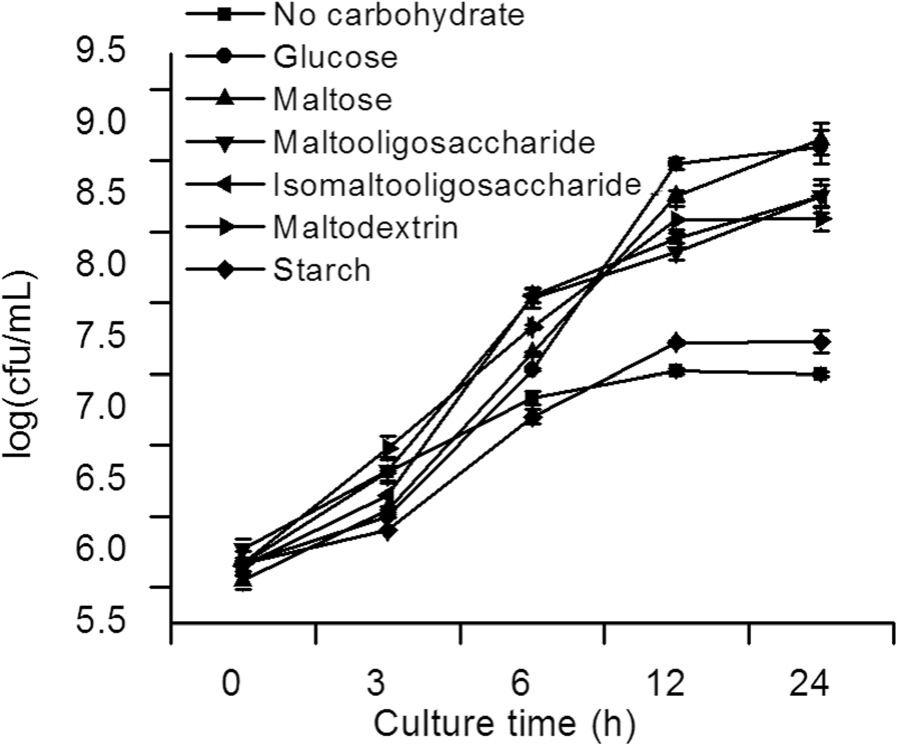
**Growth curves of BBMN68 in the presence of starch and starch hydrlysates.** Growth was measured as log cfu/ml.

The growth of BBMN68 was assessed, when starch, maltodextrins, maltooligosaccharide, isomaltooligosaccharides, maltose and glucose were used as sole carbon sources. BBMN68 showed excellent growth on all predicted substrates, reaching 10^8^ cfu/mL after 6 h (Figure 4). However, this strain could not grow well in the medium with starch as carbon source, which also confirmed the genomic analysis.

**Figure 5:**
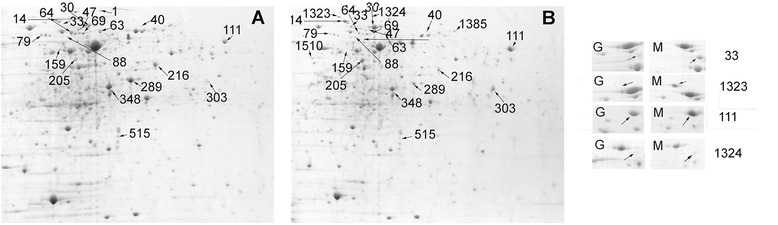
**2-D gel electrophoresis (pH4-7) of whole-cell proteins of BBMN68 grown on semisynthetic medium containing glucose (A) and maltodextrins (B).** The spots labeled on the 2-D maps were identified by MALDI-TOF/TOF analysis.

**Figure 6:**
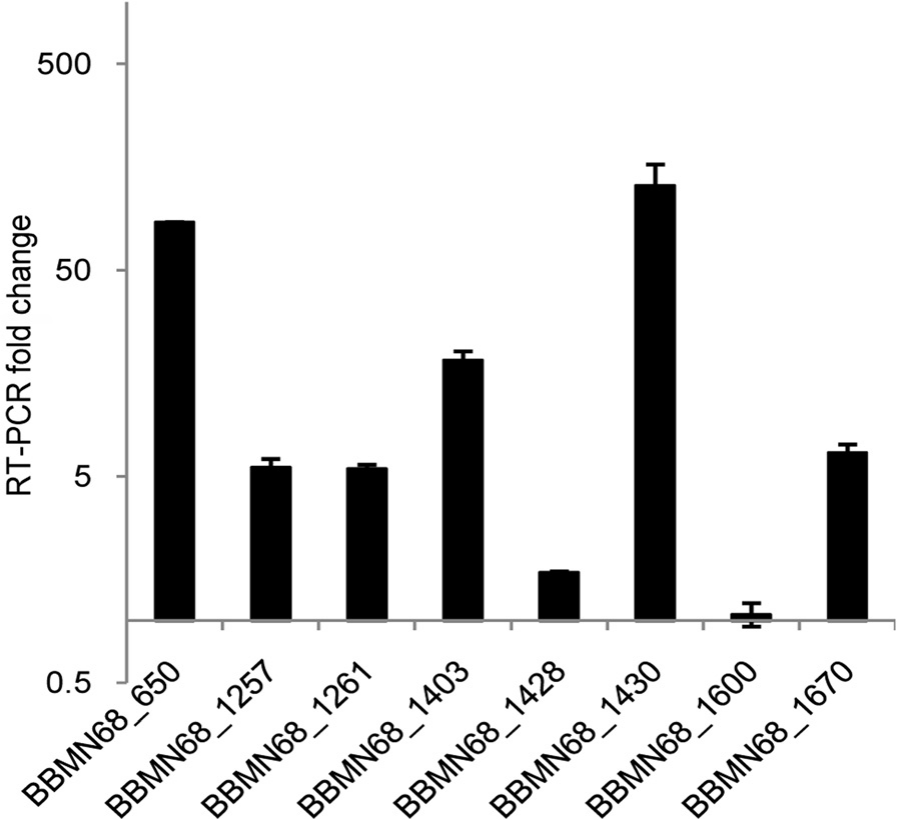
**RT-PCR analysis of mRNA expression of studied genes.**

The change of protein abundance during mid-exponential phase was analyzed using two-dimensional electrophoresis when BBMN68 was grown in the medium with maltodextrins as sole carbon source and BBMN68 grown on glucose was used as a control. These results were used to verify the predicted genes dedicated to the utilization of starch and starch hydrolysates. A total of 34 differentially abundant spots were found (Figure 5). All of these spots were identified by MALDI-TOF MS/MS (Table 1). Among them, 16 proteins were up-regulated and 16 were down-regulated by more than 2-fold. Among the 16 over-produced proteins, 4 were predicted to be involved in degrading starch and starch hydrolysates (BBMN68_1261, alpha-glucosidase; BBMN68_1430, alpha-glucosidase; BBMN68_1600, oligo-1,6-glucosidase; and BBMN68_650, alpha-amylase). In addition, 2 components of ABC-type sugar transporters (BBMN68_1403, BBMN68_1670) and a glyceraldehyde 3-phosphate dehydrogenase (BBMN68_254) showed more abundant. We then analyzed the transcription of all the genes in the predicted pathway and the two genes encoding components of the ABC-type carbohydrate transporter by RT-PCR (Figure 6). The transcription level of genes *BBMN68_1403* and *BBMN68_1670* were 22-and 7-fold up-regulated, respectively (Figure 6). *BBMN68_650*, *BBMN68_1257*, *BBMN68_1261*, *BBMN68_1428* were also up-regulated when BBMN68 was grown on medium with maltodextrins with the sole carbon source. Our results showed that the predicted genes for degrading the starch and starch hydrolysates were up-regulated at transcriptional and/or translational level.

**Table 1:** **Proteins exhibiting changed expression**

**Spot no**	Fold change*	**Predicted protein**	**Locus**	**Protein score**
69	−4.38	Glutamine synthetase 1	BBMN68_187	198
47	−3.13	Phosphoglucomutase	BBMN68_1663	239
159	−3.03	Enolase	BBMN68_771	224
382	−3.03	Pyridoxine kinase	BBMN68_682	109
289	−2.94	Ketol-acid reductoisomerase	BBMN68_1262	177
535	−2.94	COG0094: Ribosomal protein L5		161
63	−2.00	F0F1 ATPsynthase subunit alpha	BBMN68_1120	182
515	−2.50	Glutamine amidotransferase subunit PdxT	BBMN68_906	133
422	−2.38	Phosphate transport system ATP-binding protein	BBMN68_1075	296
260	−2.33	Phosphoribosylaminoimidazole (AIR) synthetase	BBMN68_870	88
79	−2.22	Saly-type abc antimicrobial peptide transport system permease component	BBMN68_1451	112
305	−2.13	4-diphosphocytidyl-2-methyl-erithritol synthase	BBMN68_1087	121
542	−2.13	Putative phosphoketolase	BBMN68_708	105
1	−2.13	GTP-binding elongation factor TypA/BipA	BBMN68_1650	85
64	−2.13	ATP synthase beta chain	BBMN68_1118	127
216	−2.08	Malate/lactate dehydrogenases	BBMN68_193	93
348	−2.00	Pyridoxine biosynthesis protein	BBMN68_907	174
88	2.18	Phosphotetolase	BBMN68_708	118
40	2.22	Inosine-5′-monophosphate dehydrogenase	BBMN68_1755	169
14	2.24	COG0539: Ribosomal protein S1 [Bifidobacteriumlongum DJO10A]	BBMN68_742	563
44	2.83	DppA2 [Bifidobacteriumlongum NCC2705]	BBMN68_277	251
303	3.84	Hypothetical protein BL1418 [Bifidobacteriumlongum	BBMN68_307	194
111	3.89	ATP binding protein of ABC transporter for sugars	BBMN68_1403	153
30	3.94	COG1621: Beta-fructosidases (levanase/invertase)	BBMN68_151	245
33	4.06	Alpha-1,4-glucosidase; maltase-like enzyme	BBMN68_1261	346
205	5.27	L-1,2-propanediol oxidoreductase [Bifidobacteriumlongum NCC2705]	BBMN68_1706	456
102	5.85	Ribosomal protein S2		183
1323	Induce&	COG0366: Glycosidases [Bifidobacteriumlongum	BBMN68_1600	86
1324	Induce	Glycosidase	BBMN68_650	167
1385	Induce	Alpha-1,4-glucosidase; maltase-like enzyme	BBMB68_1430	216
1401	Induce	Peptide chain relase factor 2	BBMN68_946	185
1510	Induce	Mutiple sugar transport systermsubstrat- binding protein	BBMN68_1670	166
1730	Induce	Phosphoribosylaminoimidazole-succinocarboxamide synthase	BBMN68_854	114
1412	5.37	GapA; gapa; K00134 glyceraldehyde 3-phosphate dehydrogenase [EC:1.2.1.12]	BBMN68_254	

*Fold change relative to control: positive values represent upregulated proteins, negative values downregulated proteins; & means that the spot is expressed specifically in cells when grown on maltodextrins.

## Discussion

Bifidobacteria are dominant in the infant gut, but they account only for 3-6% of the adult fecal flora [1]. The carbohydrates available in the gut are considered to be a main reason for this difference. In the present study, we focused on their preferred carbohydrates in human gut to elucidate the nutrient-niche of bifidobacteria. GHs participating in the degradation of starch and starch hydrolysates were more abundant in bifidobacteria than in other intestinal bacterial strains, indicating that bifidobacteria prefer to these substrates in human gut. In fact, the preference of bifidobacteria to starch and starch hydrolysates was also reported in previous studies. Bifidobacteria can selectively adhere to starch among the insoluble substrates in human gut [21]. In addition, many bifidobacterial strains have been demonstrated to be able to utilize starch [16,22]. Meanwhile, starch was more effective than other polysaccharides to increase the number of bifidobacteria in human gut [23]. Compared with bacteroidetes, which are the most extensive users of polysaccharides in human gut [24,25], bifidobacteria also have genetic advantages in the utilization of starch and starch hydrolysates. The total number of GHs in bacteroidetes is higher than that in bifidobacterial genomes. However, both the percentage and number of GHs for degrading starch and starch hydrolysates were higher in bifidobacterial genomes than in bacteroidetes genomes. A previous study also showed that the number of GHs in GH13 family was lower in *Bacteroides thetaiotomicron* than in *B.longum* [26].

Compared with other polysaccharides in the human gut, starch and starch hydrolysates are highly fermentable carbon sources for many strains [22]. By investigating GH profiles, we found that a complete starch pathway for degrading starch and starch hydrolysates also exists in other species, such as *Bacteroide*s spp, *Lactobacillus* spp. But it is worth noting that in these species, the complete starch and starch hydrolysates degrading pathway was not present in all stains. For example, 10 of all the 25 lactobacillus strains analyzed were devoid of this pathway. However, this pathway was conserved in all the 25 bifidobacterial genomes.

In the present study, predicted genes were demonstrated to be involved in the utilization of maltodextrins by proteomic and RT-PCR analysis. But caution remains necessary in extrapolating results of in vitro studies under this optimal condition to the complexity condition of the highly competitive gut environment. Until now, there was no report about the activity of a GH in the human gut. However, Motherway et al. reported that the GH genes of *B. breve* UCC2003 were induced during transit through the murine [27]. Furthermore, glycoside hydrolases in *Bac. thetaiotaomicron* were selectively induced when this strain colonized in germ-free mice feed with polysaccharide rich foods [28].

Besides starch and starch hydrolysates, some other oligosaccharides in human gut such as fructo-oligosaccharides and host-derived mucin, could also be used by bifidobacterial strains [7]. Furthermore, bifidobacteria could adhere to the mucus or intestinal epithelial cells [29,30]. Finally, genomic analysis revealed that the bifidobacterial genomes encoded higher number of transcription regulators, which could response quickly to the fluctuation of environments in human gut [1]. All these factors contribute for bifidobacteria to survive and persist in the human gut. In infant gut, HMOs were the main carbon sources, which are exclusively fermented by bifidobacterial species [31,32]. The abundant and exclusive properties of these components make the bifidobacteria predominant in infant gut. However, dietary polysaccharides, such as plant cell wall polysaccharides, are the main carbon sources in adult gut [9]. Bifidobacteria could only digest relatively lower number of these complex substrates such as starch and starch hydrolysates. In addition, the composition of adult gut microbiota is more complex than that of infant gut microbiota. The competition of other members of the gut microbiota for both nutrients and adherent sites could also be accounted for the low abundant of bifidobacteria in adult gut.

BBMN68 isolated from centenarian was incapable of growing on starch, as it lacks of extracellular amylase. A primary survey of the diet of host of BBMN68 showed that corn was the most frequently ingested staple food. Corn is one of the most common sources of resistant starch [10]. Englyst et al. reported that after ingesting of cornflakes, starch and maltodextrins were present in the lower gut [15]. It is speculated that high intake of corn can provide intestinal microbiota with available maltodextrins, which further promote growth and propagation of bifidobacteria in the gut of centenarian. Moreover, high abundance of *Bacteroides* spp. was reported in the centenarians, which have been reported to have extracellular enzymes that target a wide range of plant polysaccharides [33]. The cross-feeding by *Bacteroides* spp. provided further advantages for bifidobacteria to grow in the centenarians’ gut [34].

## Conclusions

The number of GHs for degrading starch and starch hydrolysates was higher in PBG than in RM, and they were conserved in all studied bifidobacterial strains. The pathway for degrading starch and starch hydrolysates was the only complete pathway in BBMN68 genome. Starch and starch hydrolysates were predicted to the most favorablecarbon sources of bifidobacteria. The relatively low abundance and continuous presence of these carbon sources in adult human colon were speculated to be an important reason for the relatively low but persistent abundance of bifidobacteria in the adult gut.

## Methods

### Bacterial strain and growth conditions

*Bifidobacterium longum* subsp. *longum* BBMN68 (CGMCC No. 2265, China General Microbiological Culture Collection Center ) was originally isolated from centenarians at Bama County of Guangxi Province in China. The strain isolation was approved by the Human Ethics Committee of China Agricultural University and all participants gave their consent. For culture of this strain, it was grown in Man–Rogosa–Sharpe (MRS) medium (Sharlau, Spain) supplemented with 0.05%L-cysteine-HCl at 37°C in Hungate tubes, which were initially spared with a gas of 99.99% N_2_ to maintain an anaerobic environment.

### Carbohydrate growth assay

Semisynthetic medium [35], supplemented with 1% (w/v) of a particular carbohydrates, was used. The starch, maltodextrins, maltooligosaccharide, isomaltooligosaccharides, maltose and glucose as carbon source, respectively. The semisynthetic medium consisted of 1%bactopeptone (w/v), 0.5% yeast extract (w/v), 0.2% dipotassium phosphate (w/v), 0.5% sodium acetate (w/v), 0.2% ammonium citrate (w/v), 0.02% magnesium sulfate (w/v), 0.005% manganese sulfate (w/v) and 0.1% Tween 80 (v/v). The number of colony forming unit (CFU) was monitored by plate count on MRS agar medium at 0, 3, 6, 9, 12 and 24 h, respectively.

### Bioinformatic analysis

The reference genomes areacquired under the accession number and are listed in Additional file 1. To identify the number, type, family and function of the GH genes, each genome was subjected tothe analytical pipeline used in the CAZy database [36]. The substrates of GHs are predicted as described by Brandi [19]. The protein subcellular localization of GHs was analyzed by PSORTdb [37]. Based on the GH genes of the listed bifidobacterial genomes, the BLAST heatmap was produced through NCBI blast and the corresponding similarity values were grouped using Cluster.3.0.

### Two-dimensional gel electrophoresis (2-DE)

Whole bacterial protein extracts were prepared as previouslydescribed [38]. For electrophoresis in the first dimension, total whole-cell protein (800 μg) was loaded onto the IPG strips (24 cm, pH 4-7; GE Healthcare) with 450 μl rehydration solution (7 M urea, 4% CHAPS, 50 mM DTT, 1% v/v IPG bufferpH 4-7). IEF was performed in an IPGphor system (GE Healthcare) with the following voltage gradient: 200 V for800 V. h, 500 V for 1000 V. h, from 500 to 1000 V for 800 V. h, 1000 V for 1000 V. h, from 1000 to 8000 V for 13500 V. h, 8000 V for 80000 V. h, for a total of 97.1 kVh. Following electrophoresis, image analysis, in-gel digestion and protein identification were performed as previously described by Xiao et al. [38].

### RT-PCR analysis

RNA isolation and cDNA synthesis were performed as described by Jin et al. [39]. Primer sequences were designed usingonline Primer-blast software available on the NCBI blast database and were synthesized by Invitrogen. The primers are summarized in Table 2. The RT-PCR was carried out with a Techne Quantica real-time PCR detection system (TECHNE), and 16S rDNA was used as the reference gene [40].

**Table 2:** **Primers used for real-time quantitative PCR**

**Gene ID**	**Primer sequence (F/R: 5′-3′)**	**Product size (bp)**	**Reference**
*16S*	CTGAGATACGGCCCAGACTC	279	[41]
	AAGCGATGGACTTTCACACC		
*BBMN68_650*	CGTACGTCCGAAGTTCCCCG	199	This study
	CACGGTCAGGGAATGCTGGG		
*BBMN68_1600*	CAGGATTCGAACGGGGACGG	187	This study
	CCATATCCTCGAGCGTGCCG		
*BBMN68_1261*	GCGCAACGGCACCACATATC	171	This study
	GCGGTCGGATCCTCCAAGT		
*BBMN68_1670*	GAGACCGATGGCTCCAAGGC	168	This study
	GGAGGTCATGAACAGCGGGG		
*BBMN68_1430*	GGCTCAGCGTAACGAGCACA	169	This study
	GTTCTGCACGGCAGTCTGGT		
*BBMN68_1428*	GATGTGGATCCCCGCCTTGG	157	This study
	CCGATTCCGGACCTTGAGCC		
*BBMN68_1403*	GCCTTCTCCCTGAAGGTTGT	111	This study
	GTCGGACATGACCTGGGAAG		
*BBMN68_1257*	CGTATGGGTGACCAACTGGG	147	This study
	GGCGTAGTAGTAGCCGGAGA		

## Additional files

Additional file 1:
**Bifidobacterial strains used in this study.**
Additional file 2:
**Genes encoding GHs in BBMN68 genome.**
