# Supplementary material for: Starch and starch hydrolysates are favorable carbon sources for Bifidobacteria in the human gut
Source: BMC Microbiol. 2015 Mar 1;15:54. doi: 10.1186/s12866-015-0362-3 (PMC4349234; doi:10.1186/s12866-015-0362-3)
Supplement: Additional file 2: — Genes encoding GHs in BBMN68 genome. [file 12866_2015_362_MOESM2_ESM.docx]

**Table S1 Predicted glycosy hydolylase of *B. longum* subsp. *longum* BBMN68**

| **Gene ID** | **GH family** | **EC number** | **Subcellular localization** | **Predicted enzymatic activity** | **Predicted substrates** |
| --- | --- | --- | --- | --- | --- |
| *BBMN68_1812* | GH2 | EC:3.2.1.23 | Unknown | beta-galactosidase | Plant or animal |
| *BBMN68_221* | GH3 | EC:3.2.1.21 | Cytoplasmic | beta-glucosidase | Plant/Animal |
| *BBMN68_777* | GH3 | — | Cytoplasmic | — | Plant/Animal |
| *BBMN68_1392* | GH3 | — | CytoplasmicMembrane | — | Plant/Animal |
| *BBMN68_1792* | GH3 | EC:3.2.1.21 | Cytoplasmic | beta-glucosidase | Plant/Animal |
| *BBMN68_220* | GH5 | — | Cytoplasmic | — | Plant/Fungal |
| *BBMN68_1793* | GH5 | — | CytoplasmicMembrane | beta-glucosidase | Plant/Fungal |
| *BBMN68_650* | GH13 | — | Cytoplasmic | alpha-amylase | Starch |
| *BBMN68_732* | GH13 | — | Cytoplasmic | glycogen operon protein | Starch |
| *BBMN68_749* | GH13 | — | Cytoplasmic | 1,4-alpha-glucan branching enzyme | Starch |
| *BBMN68_1127* | GH13 | EC:2.4.99.16 | Cytoplasmic | starch synthase | Starch |
| *BBMN68_1257* | GH13 | EC:3.2.1.1 | Unknown | alpha-amylase | Starch |
| *BBMN68_1258* | GH13 | — | Unknown | — | Starch |
| *BBMN68_1261* | GH13 | EC:3.2.1.20 | Cytoplasmic | alpha-glucosidase | Starch |
| *BBMN68_1267* | GH13 | EC:2.4.1.7 | Cytoplasmic | sucrose phosphorylase | Starch |
| *BBMN68_1428* | GH13 | EC:3.2.1.20 | Cytoplasmic | alpha-glucosidase | Starch |
| *BBMN68_1430* | GH13 | EC:3.2.1.10 | Cytoplasmic | oligo-1,6-glucosidase | Starch |
| *BBMN68_1600* | GH13 | EC:3.2.1.10 | Cytoplasmic | oligo-1,6-glucosidase | Starch |
| *BBMN68_1561* | GH13 | EC:2.4.1.4 | Cytoplasmic | amylosucrase | Starch |
| *BBMN68_1610* | GH13 | EC:3.2.1.- | Cytoplasmic | pullulanase-like glycosidase | Starch |
| *BBMN68_99* | GH20 | — | Cytoplasmic | lnbB | Animal/Fungal |
| *BBMN_992* | GH23 | — | Unknown | — | Petidoglycan |
| *BBMN68_1389* | GH23 | — | Unknown | — | Petidoglycan |
| *BBMN68_552* | GH25 | — | Extracellular | LysM Glyco_hydro_25 | Petidoglycan |
| *BBMN68_1471* | GH27 | — | Unknown | alpha-galactosidase | Plant |
| *BBMN68_1454* | GH30 | — | Unknown | — | Others |
| *BBMN68_1791* | GH30 | EC:3.2.1.45 | CytoplasmicMembrane | glucosylceramidase | Others |
| *BBMN68_101* | GH31 | EC:3.2.1.177 | Cytoplasmic | alpha-glucosidase | Others |
| *BBMN68_1254* | GH31 | — | Cytoplasmic | alpha-glucosidase | Others |
| *BBMN68_151* | GH32 | EC:3.2.1.26 | Cytoplasmic | beta-fructofuranosidase | Sucrose/Ohters |
| *BBMN68_1420* | GH36 | EC:3.2.1.22 | Cytoplasmic | alpha-galactosidase | Plant |
| *BBMN68_1429* | GH36 | EC:3.2.1.22 | Cytoplasmic | alpha-galactosidase | Plant* |
| *BBMN68_215* | GH38 | EC:3.2.1.24 | Cytoplasmic | alpha-mannosidase | Animal |
| *BBMN68_216* | GH38 | EC:3.2.1.24 | Cytoplasmic | alpha-mannosidase | Animal |
| *BBMN68_930* | GH42 | EC:3.2.1.23 | Cytoplasmic | beta-galactosidase | Others |
| *BBMN68_1021* | GH42 | EC:3.2.1.23 | Unknown | beta-galactosidase | Others |
| *BBMN68_1162* | GH43 | — | Cellwall | extracellular protein possibly involved in xylan or arabinan degredation | Plant |
| *BBMN68_1442* | GH43 | — | Cellwall | — | Plant |
| *BBMN68_1443* | GH43 | — | Cellwall | — | Plant |
| *BBMN68_1444* | GH43 | — | Extracellular | — | Plant |
| *BBMN68_1445* | GH43 | — | Cellwall | — | Plant |
| *BBMN68_1455* | GH43 | — | Cellwall | — | Plant |
| *BBMN68_1446* | GH43 | — |  | — | Plant |
| *BBMN68_1572* | GH43 | EC:3.2.1.55 | Extracellular | arabinoxylan | Plant |
| *BBMN68_1573* | GH43 | — | Extracellular | xynB3 | Plant |
| *BBMN68_886* | GH51 | — | Extracellular | alpha-N-arabinofuranosidase | Plant |
| *BBMN68_928* | GH51 | EC:3.2.1.55 | Cytoplasmic | alpha-N-arabinofuranosidase | Plant |
| *BBMN68_1646* | GH51 | EC:3.2.1.55 | Cytoplasmic | alpha-N-arabinofuranosidase | Plant |
| *BBMN68_1018* | GH53 | — | Cellwall | — | Plant |
| *BBMN68_1259* | GH77 | EC:2.4.1.25 | Cytoplasmic | 4-alpha-glucanotransferase | Starch |
| *BBMN68_1607* | GH77 | EC:2.4.1.25 | Cytoplasmic | 4-alpha-glucanotransferase | Starch |
| *BBMN68_222* | GH85 | — | Extracellular | endo-beta-n-acetylglucosaminidase | Animal |
| *BBMN68_1202* | GH101 | EC 3.2.1.97 | Extracellular | endo-a-N-acetylgalactosaminidase | Animal |
| *BBMN68_1673* | GH112 | — | Cytoplasmic | lacto-n-biose phosphorylase; | Others |
| *BBMN68_1163* | GH121 | — | Unknown | — | Plant |
| *BBMN68_225* | GH125 | — | Unknown | — | Others |
| *BBMN68_1167* | GH127 | — | Cytoplasmic | — | Others |
| *BBMN68_1468* | GH127 | — | Cytoplasmic | — | Others |

Plant: plant cellwall polysaccharide

Animal: animal carbohydrates

Plant/animal: CHs in this category may target on plant cellwall polysaccharides or on animal carbohydrates.

Others: carbohydrates except the what have been listed here.

Sucrose/Ohters: GHs in this category may target sucrose or fructans or others

—: no information annonted
